# LRRK2 contributes to monocyte dysregulation in Parkinson’s disease

**DOI:** 10.1186/s40478-016-0396-2

**Published:** 2016-11-24

**Authors:** Corinna Bliederhaeuser, Lisa Zondler, Veselin Grozdanov, Wolfgang P. Ruf, David Brenner, Heather L. Melrose, Peter Bauer, Albert C. Ludolph, Frank Gillardon, Jan Kassubek, Jochen H. Weishaupt, Karin M. Danzer

**Affiliations:** 1 Department of Neurology, Ulm University, Albert Einstein Allee 11, 89081 Ulm, Germany; 2 Department of Neuroscience, Mayo Clinic Jacksonville, Jacksonville, FL USA; 3 Boehringer Ingelheim Pharma GmbH & Co KG, CNS Diseases Research, Biberach/Riss, Germany

**Keywords:** LRRK2, Monocytes, Parkinson’s disease, Inflammation

Mutations in the *leucine-rich repeat kinase 2* (*LRRK2*) gene are the most common cause of familial Parkinson’s disease (PD) [20, 32]. Common polymorphisms in *LRRK2* have been shown to modulate the risk for sporadic PD [6, 23, 24] strengthening the idea that inherited and sporadic PD share common underlying pathways. Although LRRK2 has been associated with a variety of cellular functions, including autophagy [1], mitochondrial function/dynamics [30] and microtubule/cytoskeletal dynamics [12], the overall physiological function of LRRK2 and its role in PD are only partially understood. Relatively recent studies also support a role for LRRK2 as regulator of inflammation. Substantial levels of LRRK2 protein and mRNA have been reported in immune cells like peripheral blood mononuclear cells (PBMCs), including B-cells, monocytes/macrophages, and dendritic cells [9, 13, 16, 29]. Moreover, LRRK2 has been shown to be involved in the activation and maturation of immune cells [29], in controlling the radical burst against pathogens in macrophages [9] and in modulating neuroinflammation by cytokine signaling [10, 19]. Remarkably, elevated levels of serum cytokines (IL-2, IL-4, IL-6, IL-10, TNFα) in PD patients [4, 22, 27] point to an involvement of the peripheral immune system in the pathogenesis of PD. Recently, we found an enrichment of “classical” CD14^++^CD16^−^ monocyte subpopulation in the peripheral blood of PD patients together with a dysregulation of inflammatory pathways, phagocytosis deficits as well as hyperactivation of PD monocytes in response to LPS treatment, which correlated to PD severity [11]. Here, we sought to study the contribution of LRRK2 to the dysregulation of monocytes in Parkinson’s disease.

We set out to obtain a comprehensive picture of LRRK2 levels in circulating monocyte subpopulations as well as in lymphoid B-cells in PD patients. To determine the intracellular LRRK2 protein levels in the different immune cells we established a flow cytometry-based technique for intracellular LRRK2 staining. To verify the specificity of the anti-LRRK2 antibody used in this study [Novus Biologicals (NB300-268AF647)] isolated murine spleen cells from LRRK2 knockout (KO) mice [14] and mice overexpressing human wild-type (WT) LRRK2 (LRRK2 WT-OX mice) [17, 28] were processed, stained and analyzed as described in the supplementary material and method section (Additional file 1). While we found a highly LRRK2-positive population with the Novus antibody in spleen samples of LRRK2 WT-OX mice (black histogram Fig. 1a) no unspecific staining was found in LRRK2 KO mice (dark grey histogram Fig. 1a) or with the isotype control (IgG ctrl.) in spleen samples of LRRK2 WT-OX mice (light grey histogram Fig. 1a).

**Fig. 1:**
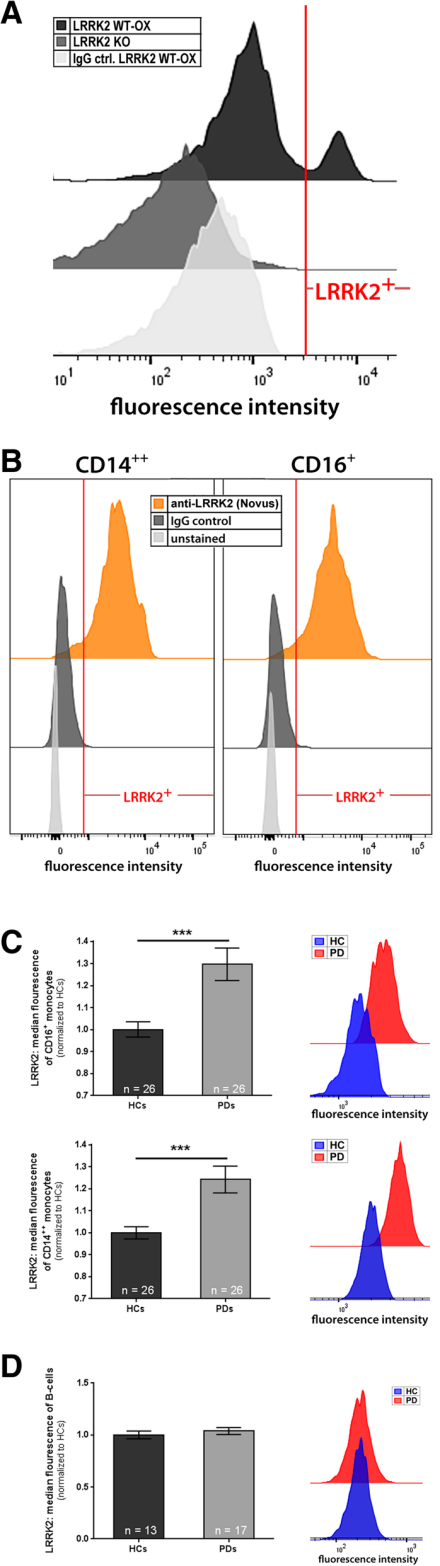
LRRK2 protein expression is significantly upregulated in monocytes from PD patients. **a** Spleen cells from LRRK2 KO and LRRK2 WT-OX mice were used to validate the suitable application of the rabbit-anti-LRRK2 antibody conjugated to AlexaFluor®647 from Novus Biologicals (NB300-268AF647) for intracellular flow cytometry analyses. The antibody showed a highly positive LRRK2 population in LRRK2 WT-OX cells (black histogram), whereas no LRRK2 staining was presented within KO cells (dark grey histogram), nor in LRRK2 WT-OX cells stained with the monoclonal rabbit isotype control (light grey histogram). The displayed experiment shows the fluorescence intensity of the different samples and is representative of three independent experiments. **b** Further validation experiments of intracellular LRRK2 staining for FACS analyses were performed with human whole blood samples. The human CD14^++^ and CD16^+^ monocyte subpopulations showed positive staining for LRRK2 [anti-LRRK2 (Novus); orange histogram], while the isotype control staining did not show any nonspecific binding (‘IgG control’; dark grey histogram). The displayed graphs are representative of three independent experiments. **c** Leukocytes from whole blood samples of healthy controls (HC; *n* = 26) and PD patients (PD; *n* = 26) were analyzed by flow cytometry to detect LRRK2 protein in the different monocyte subsets. CD16^+^ monocytes (upper panel) as well as CD14^++^ monocytes (lower panel) from PD patients displayed significantly higher LRRK2 expression compared to respective monocyte subsets from healthy controls. The histograms on the right are representative for the analyzed individuals and display the fluorescence intensity of the anti-LRRK2-AlexaFluor®647 antibody. The higher LRRK2 expression in monocytes of PD patients compared to healthy controls is demonstratively shown in these graphs. **d** Flow cytometric analyzes of CD19^+^ B-cells reveal no changes in the LRRK2 protein expression between healthy controls (*n* = 13) and PD patients (*n* = 17). The histograms on the right hand side represent the fluorescence intensity of the anti-LRRK2-AlexaFluor®647 antibody and show overlapping peaks which reveal no differences in LRRK2 expression. Error bars represent mean ± SEM; **p < 0.01; statistical significance was tested with non-parametric testing

We used a combination of our well established five-color FACS analysis strategy [5, 11] to distinguish “classical” CD14^++^CD16^−^ (hereinafter referred to as CD14^++^) monocytes and “non-classical” CD14^dim^CD16^+^ (hereinafter referred to as CD16^+^) monocytes together with the intracellular LRRK2 staining. We found that both monocyte subpopulations were LRRK2 positive (orange histograms Fig. 1b), while the IgG and unstained controls did not show any significant staining (dark and light grey histograms Fig. 1b). Strikingly, we found significantly higher LRRK2 protein levels in CD16^+^ monocytes (upper panel Fig. 1c) as well as in CD14^++^ monocytes (lower panel Fig. 1c) of PD patients (*n* = 26; mean age 71.0 years) compared to age- and sex matched healthy controls (*n* = 26; mean age 68.7 years). Of note, probands with confounding factors affecting the immune system were excluded from all experiments (a detailed description of the proband cohort can be found in Additional file 1: Table S1). Similar results of elevated LRRK2 levels in monocytes from PD patients were also confirmed with two additional monoclonal anti-LRRK2 antibodies from abcam (MJFF5 (68–7) and UDD3 30(12)) (Additional file 2).

Since monocytes only represent ~ 6–12% of PBMCs [2] we also asked whether endogenous LRRK2 levels are altered in the remaining cell population predominantly comprising lymphocytes. T cells are devoid of LRRK2 [29], we thus focused on studying LRRK2 levels in B-cells of PD patients and healthy controls. Of note, we observed a significant reduction in the number of B-cells which has also been described earlier [21, 26] (data not shown). However, as demonstrated in Fig. 1d we did not detect altered LRRK2 protein levels in CD19^+^ B-cells between PD patients (*n* = 13; mean age 68.1 years) and controls (*n* = 17; mean age 71.4 years), indicating that LRRK2 levels are specifically increased in monocytes of PD patients. Our findings may explain previous results showing no increase in LRRK2 protein levels in PD patients’ PBMCs [7] since the increase of LRRK2 protein in monocytes may be masked by unchanged LRRK2 levels in B-cells.

Previously, we have shown a dysregulation of monocyte subpopulations in the peripheral blood of PD patients [11]. Having now found that LRRK2 levels are elevated in PD monocytes, we next asked whether this LRRK2 increase might be involved in monocyte subtype dysregulation. Consequently, we assessed monocyte subpopulations in a LRRK2^(R1441G)^ BAC transgenic mouse model overexpressing the mutant form of human LRRK2, recapitulating main features of Parkinson’s disease [17]. In mice CD14^++^ monocytes correspond to Ly6C^high^ monocytes and CD16^+^ monocytes to Ly6C^low^ monocytes [15]. Using six-color flow cytometry [3, 8] LRRK2^(R1441G)^ mice showed a marked age-dependent increase in the ratio of Ly6C^high^ to Ly6C^low^ monocytes (Fig. 2a) compared to non-transgenic littermates which was most prominent at 20 month of age. Also in LRRK2 WT-OX mice we found a trend for an increase in the ratio of Ly6C^high^ to Ly6C^low^ monocytes, although statistical significance was not reached (Fig. 2b). To additionally control that LRRK2 overexpression was persistent in PBMCs from LRRK2 WT-OX mice we performed RT-PCR and found a robust human LRRK2 expression in PBMCs from LRRK2 WT-OX mice but not in non-transgenic (NT) littermates (Fig. 2c), further strengthening the contribution of LRRK2 in shifting monocyte subpopulations in LRRK2 overexpressing mice.

**Fig. 2:**
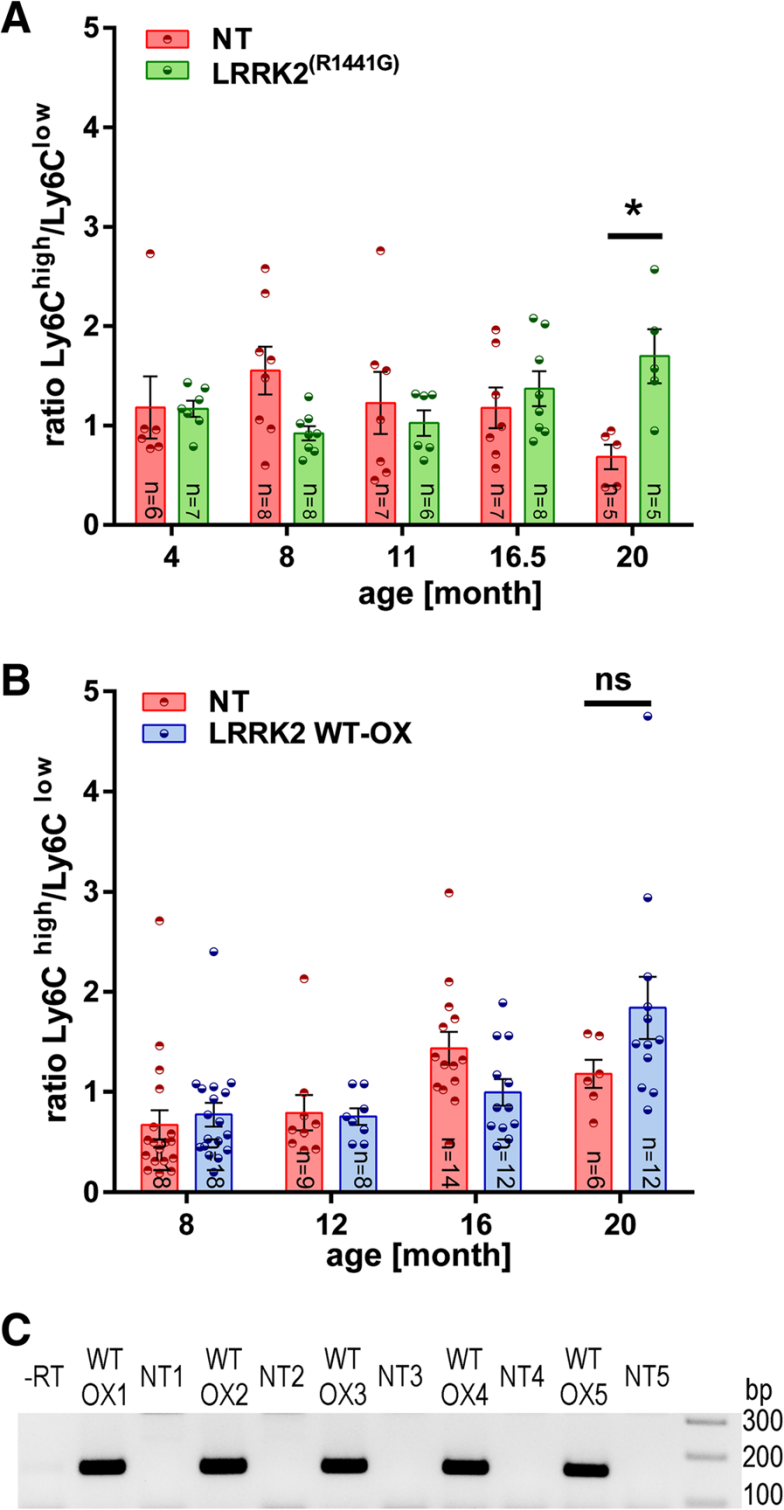
Differences in the monocyte subset ratio of human LRRK2 overexpressing mice. **a, b** Ly6C^high^ and Ly6C^low^ monocyte subsets of mouse models for PD were analyzed by six-color flow cytometry. **a** A significant increase in the ratio of Ly6C^high^ to Ly6C^low^ monocyte subsets was detected in 20 month old mutant LRRK2^(R1441G)^ BAC transgenic mice in comparison to NT littermates. **b** A trend of an increasing ratio of Ly6C^high^ to Ly6C^low^ monocytes of LRRK2 WT-OX mice compared to NT littermates were identified in 20 month old animals. Error bars represent mean ± SEM, *p < 0.05; ns: not significant; statistical significance was tested with 2way ANOVA (**c**) Murine PBMCs were isolated from whole blood samples and isolated RNA was transcribed into cDNA. PCR products were visualized on a 2% agarose-gel. A band with 153 bp represent human LRRK2 and is only detected in PBMCs from LRRK2 WT-OX mice and not in NT littermates; −RT: negative reverse transcription control, NT: non-transgenic

In summary, we found elevated LRRK2 levels in CD14^++^ and CD16^+^ monocyte subsets of PD patients, but not in patients’ B-cells. Furthermore, similar to the dysregulation of monocyte subpopulations found in PD patients [11], a dysregulation of monocyte subpopulations was detected in LRRK2 overexpressing mice. Our results add to the growing body of evidence that LRRK2 plays an important role not only in neuronal cells but also in immune cells. LRRK2 has been implicated in aspects of monocyte function including monocyte maturation [29], adhesion, migration, and inflammation [18, 19]. Furthermore, LRRK2 is hierarchically clustered in the tyrosine-kinase like superfamily nearby kinases that are important for inflammatory signaling in immune activation [31]. Moreover, LRRK2 is supposed to function as a stress response kinase since inhibition of LRRK2 in innate immune cells attenuates pro-inflammatory signaling in response to TLR4 activation [19]. During bacterial phagocytosis, LRRK2 translocates near bacterial membranes, and knockdown of *LRRK2* interrupts ROS production during phagocytosis and diminishes destruction of intracellular bacteria [9]. LRRK2 is not only found in different immune cells but becomes further upregulated upon exposure to different pathological stimuli like interferon γ (IFNγ) [9], microbial structures [lipopolysaccharide (LPS)] [10, 13] or viral particles [13]. Our current observation that LRRK2 levels are elevated in monocytes of PD patients establishes a compelling link between a specific role of LRRK2 in immune cells and their contribution to PD pathogenesis. Together with the recent study by Speidel et al. demonstrating a reduction in the non-classical CD14^+^CD16^+^ monocyte subpopulation in PD LRRK2 mutant cells [25] our study forms strong evidence for the involvement of LRRK2 in PD monocyte dysregulation. Our current study also supports the idea that PD monocytes are in a pro-inflammatory predisposition as described earlier [11] and it might be that together with the co-occurrence of “second hits” like environmental cues or CNS factors triggering the peripheral immune system LRRK2 might be upregulated in monocytes. Together with our findings on a LRRK2-dependent dysregulation of monocytes in a PD mouse model, these results strengthen the idea of a central role of LRRK2 in immune cells and its contribution in peripheral inflammation in PD. Clearly, more studies are needed to determine the role of elevated LRRK2 levels in PD monocytes, its role in dysregulation of monocyte subpopulations and in modulating inflammatory cytokine production. Moreover, the signaling pathways and the pathogenic stimulus actually leading to LRRK2 upregulation need to be determined. Our findings establish a basis for future studies on LRRK2-dependent monocyte dysregulation, peripheral inflammation and its contribution to PD pathogenesis.

## Additional files

Additional file 1: Supplementary materials and methods. (PDF 1185 kb)

Additional file 2: LRRK2 protein expression is significantly upregulated in monocytes from PD patients. (PDF 303 kb)

## Abbreviations

HC: Healthy control
IFNγ: Interferon γ
KO: Knock out
LPS: Lipopolysaccharide
LRRK2: Leucine-rich repeat kinase 2
NT: Non-transgenic
OX: Overexpressing
PBMCs: Peripheral blood mononuclear cells
PD: Parkinson’s disease
RKU: Universitäts- und Rehabilitationskliniken Ulm
WT: Wild type
