# Supplementary material for: LRRK2 contributes to monocyte dysregulation in Parkinson’s disease
Source: Acta Neuropathol Commun. 2016 Nov 24;4:123. doi: 10.1186/s40478-016-0396-2 (PMC5123218; doi:10.1186/s40478-016-0396-2)
Supplement: Supplementary file 1 — Supplementary materials and methods. (PDF 1185 kb) [file 40478_2016_396_MOESM1_ESM.pdf]

LRRK2 contributes to monocyte dysregulation in Parkinson's disease

Corinna Bliederhaeuser[1], Lisa Zondler[1], Veselin Grozdanov[1], Wolfgang P. Ruf[1], David Brenner[1], Heather L. Melrose[2], Peter Bauer[2], Albert C. Ludolph[1], Frank Gillardon[3], Jan Kassubek[1], Jochen H. Weishaupt[1], Karin M. Danzer[1,§]

[1] Department of Neurology, Ulm University, Ulm, Germany

[2] Department of Neuroscience, Mayo Clinic Jacksonville, Jacksonville, FL, USA

[3] Boehringer Ingelheim Pharma GmbH & Co KG, CNS Diseases Research, Biberach/Riss, Germany

[§]Corresponding author: Prof. Karin M. Danzer
Department of Neurology
Ulm University
Albert Einstein Allee 11
89081 Ulm, Germany
karin.danzer@uni-ulm.de
phone: +49 731-500-63049 (office)
fax: +49 731-500-63050

Additional file 1

Supplementary materials and methods

Human samples

Healthy controls were chosen to match the patient cohort's characteristics. In general, probands with confounding factors affecting the immune system were excluded from all experiments. Additionally, we controlled that anti-inflammatory drugs did not have any confounding or influencing effect on the data. All PD patients included in the study were diagnosed using the UK PD Society Brain Bank clinical diagnostic criteria at the RKU, which is a specialized center for PD. Using a standard procedure, peripheral venous blood was collected with a Monovette™ blood drawing system and processed within 3 h post collection. Li-Heparin monovettes were used for the collection of whole blood for flow cytometry analyses.

A detailed table of all probands participated in our studies can be found below.

SUPPLEMENTARY TABLE 1. Characteristics of PD patients and healthy controls for analysis of LRRK2 protein expression in monocyte subpolulations and CD19+ B cells (cohort Fig. 1 & Additional File 2)

ID	gender	age	age of onset	disease duration [y]	medication	co-morbidities	A	B	C
PD1	m	79	38	41	L-dopa, DA agonist, COMT inhibitor, NMDA antagonist	prostate hyperplasia	x		x
PD2	f	93	N/K	N/K	DA agonist, AChE inhibitor, acetylsalicylic acid	none	x		x
PD3	m	77	N/K	N/K	N/K	N/K	x		x
PD4	m	79	66	13	L-dopa, DA agonist, AChE inhibitor	prostate carcinoma (> 6 years ago), encephalopathy, depression	x		x
PD5	f	57	47	10	L-dopa, DA agonist, MAO inhibitor, ibuprofen	adiposity, arterial hypertension	x		x
PD6	m	77	69	8	L-dopa, DA agonist, COMT inhibitor	edema of the leg, borreliosis (<9 years ago)	x		x
PD7	m	74	72	2	L-dopa	encephalopathy, rectum carcinoma, depression, osteoporosis	x		x
PD8	f	64	58	6	L-dopa, DA agonist	depression, normal tension glaucoma, cataract	x		x
PD9	m	68	66	2	L-dopa, DA agonist, acetylsalicylic acid	epileptic attacks	x		x
PD10	f	68	50	18	L-dopa, DA agonist	myopathy, dizziness, spinal disc herniation, arterial hypertension	x		x
PD11	m	68	62	6	L-dopa, DA agonist, MAO inhibitor, COMT inhibitor	anxiety	x		x
PD12	m	85	78	7	L-dopa, COMT inhibitor, acetylsalicylic acid	encephalopathy, diabetes mellitus type II, arterial hypertension	x		x
PD13	f	69	N/K	N/K	L-dopa, DA agonist, MAO inhibitor, COMT inhibitor	none	x		x
PD14	m	46	41	5	DA agonist, MAO inhibitor	none	x		x
PD15	f	81	71	10	L-dopa, DA agonist, NMDA antagonist, COMT inhibitor	Sjögren's syndrome, polyneuropathy, aortic insufficiency	x		x
PD16	m	67	54	13	L-dopa, DA agonist, COMT inhibitor, NMDA antagonist	diabetes mellitus type II, hypothyroidism	x		x
PD17	f	84	75	9	L-dopa, DA agonist, MAO inhibitor, COMT inhibitor, acetylsalicylic acid	encephalopathy, arterial hypertension	x		x
PD18	m	64	56	8	DA agonist	none	x		
PD19	m	81	65	16	L-dopa, MAO inhibitor, COMT inhibitor	myopathy, degenerative lumbar spine changes, incontinence	x		

PD20	m	75	61	14	L-dopa, MAO inhibitor, AChE inhibitor, COMT inhibitor	dementia, hallucinations	x		
PD21	f	71	59	12	L-dopa, DA agonist, MAO inhibitor, COMT inhibitor	hallucinations, dyskinesia	x	x	
PD22	m	53	52	1	DA agonist	none	x	x	
PD23	f	43	38	5	DA agonist, MAO inhibitor	none	x	x	
PD24	m	73	65	8	L-dopa, DA agonist, MAO inhibitor,COMT inhibitor	diabetes mellitus type II, arterial hypertension	x	x	
PD25	m	80	69	11	L-dopa	prostate hyperplasia, dementia	x	x	
PD26	f	69	50	19	L-dopa, MAO inhibitor	dizziness, osteoporosis	x	x	
PD27	m	73	70	3	DA agonist, acetylsalicylic acid	incomplete paraplegia		x	
PD28	m	62	59	3	L-dopa, DA agonist,COMT inhibitor	diabetis mellitus type II		x	
PD29	f	68	49	19	L-dopa, MAO inhibitor	dizziness, osteoporosis		x	
PD30	m	76	66	10	L-dopa, DA agonist, MAO inhibitor	none		x	
PD31	m	78	N/K	N/K	L-dopa, clopidogrel	prostata hyperplasia, encephalopathy		x	
PD32	m	79	70	8	L-dopa, DA agonist, rivastigmine, acetylsalicylic acid	arterial hypertension, diabetis mellitus typ II		x	
PD33	m	76	N/K	N/K	L-dopa, DA agonist, acetylsalicylic acid	arterial hypertension, osteoporosis, prostate hyperplasia		x	
PD34	f	60	47	13	L-dopa, MAO inhibitor, DA agonist, COMT inhibitor	dyskinesia		x	
PD35	m	76	66	10	L-dopa, DA agonist, MAO inhibitor	none		x	
PD36	m	79	74	5	L-dopa, DA agonist, MAO inhibitor, COMPT inhibitor, clopidogrel	hyokinesia, ecephalopathy		x	
PD37	m	56	55	1	DA agonist	none		x	
PD38	m	72	61	11	L-dopa	hallucinations		x	
Ctrl.1	f	75	N/A	N/A	N/A	N/A	x		x
Ctrl.2	m	72	N/A	N/A	N/A	N/A	x		x
Ctrl.3	f	47	N/A	N/A	N/A	N/A	x		x
Ctrl.4	m	79	N/A	N/A	N/A	N/A	x		x
Ctrl.5	f	75	N/A	N/A	N/A	N/A	x		x
Ctrl.6	f	58	N/A	N/A	N/A	N/A	x		x
Ctrl.7	m	68	N/A	N/A	N/A	N/A	x		x
Ctrl.8	m	63	N/A	N/A	N/A	N/A	x		x
Ctrl.9	f	71	N/A	N/A	N/A	N/A	x		x
Ctrl.10	f	58	N/A	N/A	N/A	N/A	x		x
Ctrl.11	f	71	N/A	N/A	N/A	N/A	x		x
Ctrl.12	m	75	N/A	N/A	N/A	N/A	x		x
Ctrl.13	f	73	N/A	N/A	N/A	N/A	x		x
Ctrl.14	m	65	N/A	N/A	N/A	N/A	x		

Ctrl.15	f	65	N/A	N/A	N/A	N/A	x		
Ctrl.16	f	62	N/A	N/A	N/A	N/A	x		
Ctrl.17	m	76	N/A	N/A	N/A	N/A	x		
Ctrl.18	f	55	N/A	N/A	N/A	N/A	x	x	
Ctrl.19	m	43	N/A	N/A	N/A	N/A	x	x	
Ctrl.20	f	77	N/A	N/A	N/A	N/A	x	x	
Ctrl.21	m	68	N/A	N/A	N/A	N/A	x	x	
Ctrl.22	f	83	N/A	N/A	N/A	N/A	x	x	
Ctrl.23	m	81	N/A	N/A	N/A	N/A	x	x	
Ctrl.24	f	79	N/A	N/A	N/A	N/A	x	x	
Ctrl.25	m	71	N/A	N/A	N/A	N/A	x	x	
Ctrl.26	f	76	N/A	N/A	N/A	N/A	x	x	
Ctrl.27	f	71	N/A	N/A	N/A	N/A		x	
Ctrl.28	f	50	N/A	N/A	N/A	N/A		x	
Ctrl.29	m	78	N/A	N/A	N/A	N/A		x	
Ctrl.30	m	72	N/A	N/A	N/A	N/A		x	
Ctrl.31	f	61	N/A	N/A	N/A	N/A		x	
Ctrl.32	m	58	N/A	N/A	N/A	N/A		x	
Ctrl.33	f	76	N/A	N/A	N/A	N/A		x	
Ctrl.34	m	78	N/A	N/A	N/A	N/A		x	
Ctrl.35	f	49	N/A	N/A	N/A	N/A		x	

The table summarizes the characteristics of PD patients and healthy controls. N/A=not applicable; N/K= not known; DA=dopamine; MAO=monoaminooxidase; AChE= acetylcholinesterase; COMT=catechol-O-methyl transferase; NMDA=N-Methyl-D-aspartate; A=samples used for monocyte analysis (Novus antibody); B=samples used for monocyte analysis (abcam antibodies); C=samples used for B-cell analysis (Novus antibody)

Murine samples

LRRK2 WT-OX FVB/N mice were acquired from 'The Jackson Laboratories' (Bar Harbor, ME, USA) [20,21] and non-transgenic (NT) females were bred with heterozygous males to generate heterozygous animals and NT littermates on a FVB/N background. Mice were housed in the in-house facility of the University of Ulm with ad libitum access to food and water and a standardized light/dark cycle (14 h light/10 h dark) in a pathogen-free environment (according to FELASA) in open-top cages. Genotyping was performed with tail DNA according to the protocol provided by The Jackson Laboratory.

LRRK2^{R1441G} BAC transgenic FVB/N mice were acquired from 'The Jackson Laboratories' (Bar Harbor, ME, USA) [20,21] and NT females were bred with heterozygous males to generate heterozygous animals and NT littermates on a FVB/N background. Mice were housed in the in-house facility of Boehringer Ingelheim Biberach, Germany with ad libitum access to food and water and a standardized light/dark cycle (12 h light/12 h dark) in a pathogen-free environment in individually ventilated cages. NT females were bred with heterozygous males to generate heterozygous animals and NT littermates on a FVB/N background. Genotyping was performed with tail DNA according to the protocol provided by The Jackson Laboratory.

Flow cytometry

Human monocytes Peripheral venous blood was collected from PD patients and healthy controls with a Monovette™ blood drawing system (Sarstedt) and processed within 3 hours post collection. Li-Heparin was used as anticoagulant.

Monocyte phenotyping was performed as described previously [18,22]. Briefly, red-blood cells (RBC) were lysed (50 mM NH4Cl, 70 mM NaHCO3, 0,1% EDTA, dH2O; 10 min), two times washed in PBS + 10 % FCS, and the remaining leukocytes were blocked with Fc-block (1:20, BioLegend; 20 min). Cells were stained with anti-human antibodies specific for HLA-DR (1:200; BioLegend, L243, Pe-Cy7), CD14 (1:200; BD, M5E2, PerCP-Cy5.5), CD16 (1:10; BD, 3G8, PE), CD2 (1:20; eBioscience, RPA-2.10, FITC), CD335 (1:100; AbDSerotec, 9E2, FITC), CD15 (1:13; Miltenyi Biotec, VIMC6, FITC) and CD19 (1:100; eBioscience, HIB19, FITC) for 25 min at 4°C in the dark. For intracellular LRRK2 staining the 'Two-step protocol for intracellular (cytoplasmic) proteins' from eBioscience was followed and IC Fixation as well as permeabilization buffer was purchased from eBioscience. LRRK2 protein was detected using polyclonal rabbit anti-LRRK2-AlexaFluor®647 antibody (1.5 µg per 100 µl permeabilization buffer) from Novus Biological and data were acquired on a LSRII (BD). The two additional monoclonal anti-LRRK2 antibodies from abcam (MJFF5 (68-7) and UDD3 30(12)) used to validate intracellular LRRK2 staining in human monocytes (see Additional file 2) were used at concentrations of 1.6 µg (MJFF5) and 3.7 µg (UDD3) per 100 µl permeabilization buffer. The monoclonal rabbit IgG antibody (abcam [EPR25A]) was used as isotype control and was always used at the same concentration as the LRRK2 antibodies. Since only the Novus antibody is conjugated to AlexaFluor®647, the staining with the MJFF5, UDD3 and IgG antibodies was followed by donkey anti-rabbit IgG (clone Poly4064) AlexaFluor®647 from BioLegend (0.4 µg per 100 µl permeabilization buffer). The applied gating strategy can be seen below (Suppl. Fig. 1). Median fluorescence of LRRK2-Alexa®Fluor647 intensity was used to determine relative protein levels and data were normalized to healthy controls.

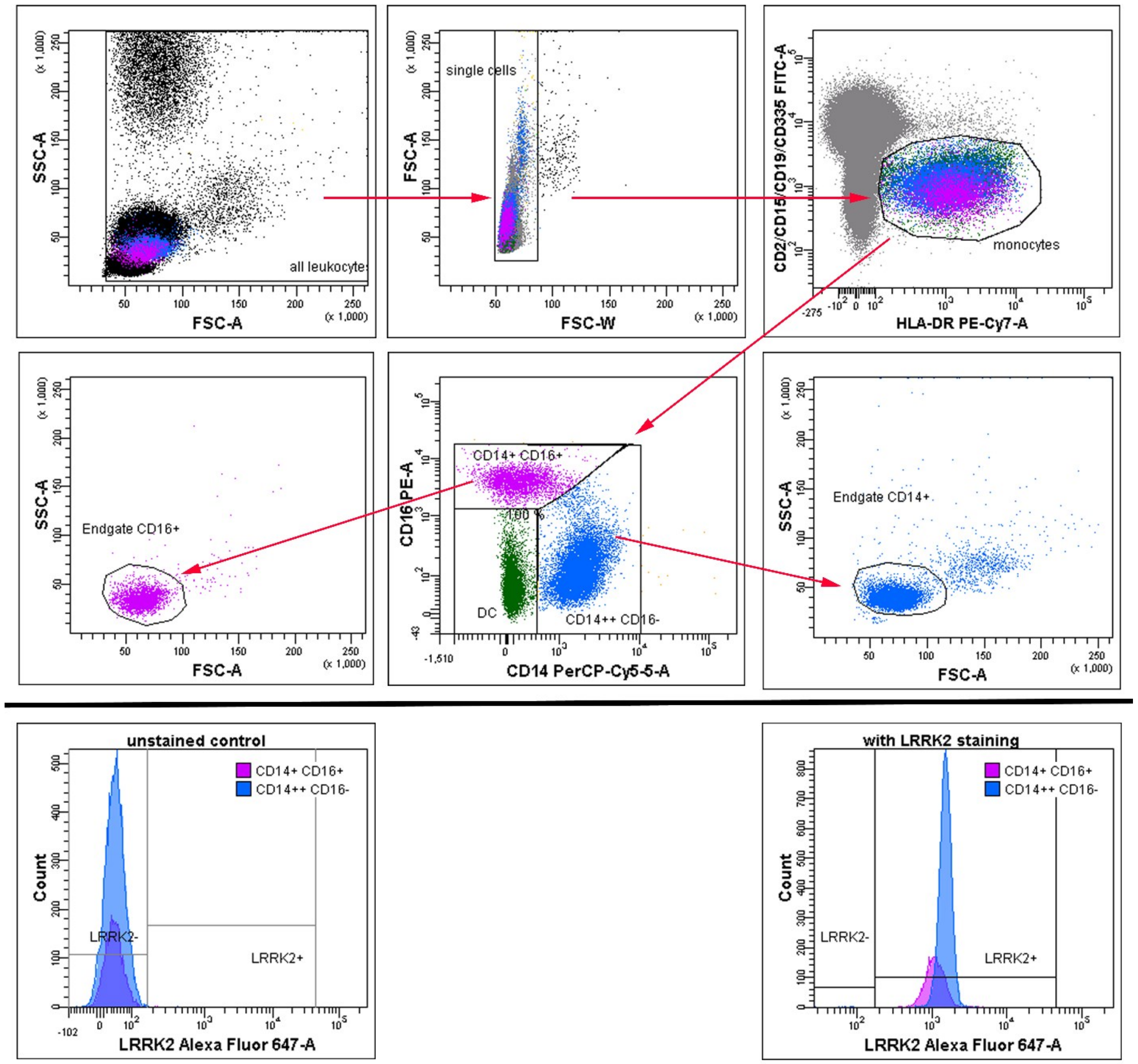

Suppl. Fig. 1 Gating strategy for flow cytometric analysis of human monocyte subpopulations and intracellular LRRK2 analyses.

Representative flow cytometric analysis of monocyte subpopulations from fresh whole blood samples after red blood cell lysis. In the first gate all living leukocytes were included and only small cell debris excluded. Next, erroneous data of cell doublets passing the laser beam were excluded by gating cells using area (FSC-A) versus width (FSC-W) signal intensity. Monocytes were then gated out of other immune cells (FITC negative: $CD2^-$, $CD15^-$, $CD19^-$ and $CD335^-$) by positive HLA-DR staining (PE-Cy7 positive). Monocyte subpopulations were characterized based on CD14 (PerCP-Cy5.5) and CD16 (PE) staining. Since monocytes have a specific size and granularity, a final gate was added to surround the respective monocyte subpopulations dependent on the forward and sideward scatter to exclude large granulocytes (which also stain positive for CD14 and CD16) or small lymphocytes from the further analysis. LRRK2 (AlexaFluor®647) intensity in $CD16^+$ and $CD14^{++}$ monocyte subsets was determined via the respective histograms and statistics from median fluorescence intensities were used. The median fluorescence intensity of the unstained control of each individual was subtracted from the measured value of the stained sample in the final analysis. At least 10,000 counts of the HLA-DR positive monocyte population were recorded for these analyses

B-cell analyzes was performed similarly as described above. After RBC lysis and leukocyte blocking, cells were stained with the anti-human CD19 antibody. Intracellular LRRK2 was stained as described above. The gating strategy for CD19$^+$ B-cells is shown below (Suppl. Fig. 2).

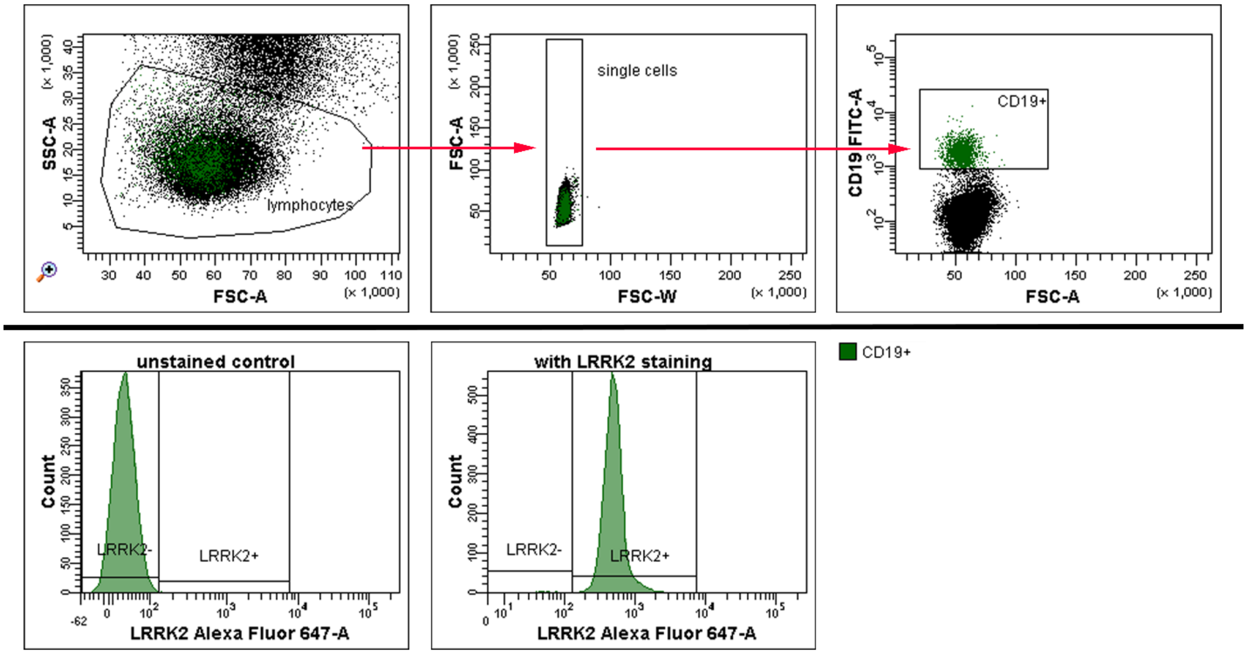

Suppl. Fig. 2 Gating strategy for flow cytometric analysis of human B-cells and intracellular LRRK2 staining. Representative flow cytometric analysis of CD19$^+$ B-cells from fresh whole blood samples after red blood cell lysis. In the first gate all living lymphocytes were included via FSC versus SSC signaling. Next, erroneous data of cell doublets passing the laser beam were excluded by gating cells using area (FSC-A) versus width (FSC-W) signal intensity. CD19$^+$ (FITC positive) B-cells were determined via FITC versus FSC gating. LRRK2 (AlexaFluor®647) intensity in B-cells was determined via the respective histograms and statistics from median fluorescence intensities were used. The median fluorescence intensity of the unstained control of each individual was subtracted from the measured value of the stained sample in the final analysis.

Murine monocytes Heterozygous LRRK2$^{(R1441G)}$ BAC transgenic as well as LRRK2 WT-OX BAC FVB/N mice were acquired from the Jackson Laboratory (Bar Harbor, ME, USA). Blood was taken from the vena facialis by puncture with a 5 mm lancet (Goldenrod) and collected in a tube rinsed with Heparin-Natrium-25000 (ratiopharm). Monocyte phenotyping was performed as described previously [28]. Briefly, RBC lysis was performed as described above and cells were blocked with TruStain Fc-block (1:50, BioLegend; 20 min) and stained (25 min, 4°C in the dark) with anti-mouse antibodies specific for NK1.1 (1:100, BD, PK136, FITC), Ly6G (1:200, BD, 1A8, FITC), CD3e (1:200, BD, 145-2C11, FITC), CD19 (1:200, BioLegend, 6D5, FITC), CD43 (1:200, BD, S7, PE), Ly6C (1:200, BioLegend, HK1.4, PerCP-Cy5.5), CD11b (1:200, BD, M1/70, APC), CD45 (1:200, BD, 30-F11, APC-Cy7) and CD115 (1:50, eBioscience, AFS98, Biotin). Cells were washed and incubated with secondary Streptavidin-PE-Cy7 antibody (1:67, eBioscience; 25 min, 4°C), 20 min fixed with 2% PFA, filtered (70 µm mesh) and analyzed by flow cytometry (LSRII, BD) using appropriate color compensation. The gating strategy can be seen below (Suppl. Fig. 3).

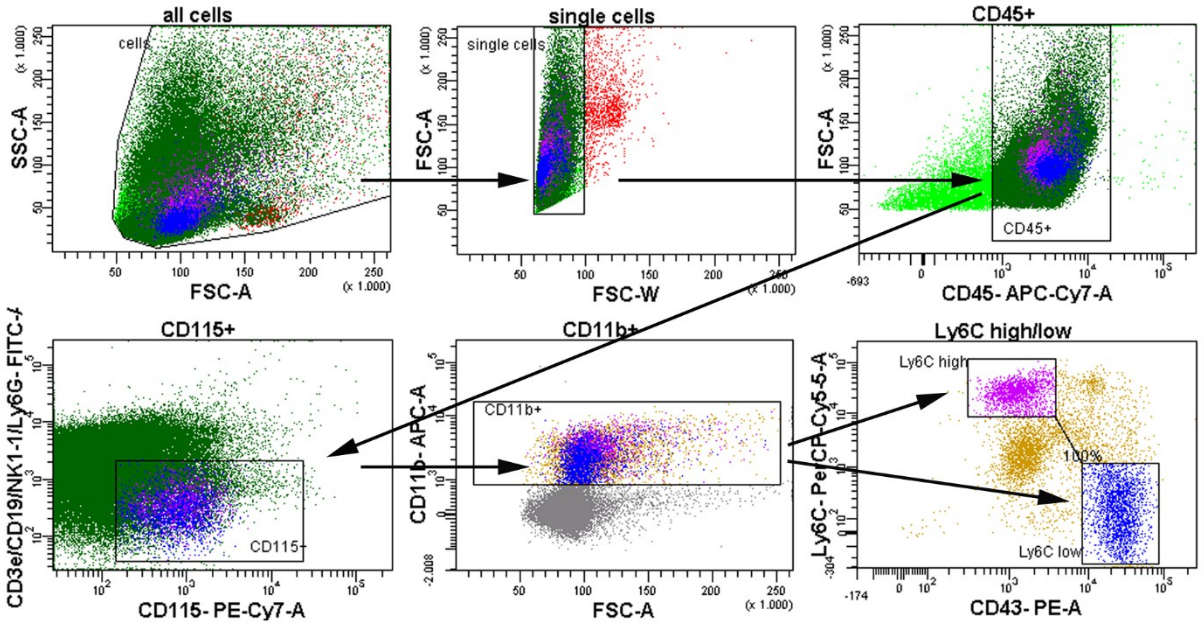

Suppl. Fig. 3 Gating strategy for flow cytometric analysis of murine monocyte subpopulations.

Representative analysis by six-color flow cytometry of monocyte subpopulations from freshly isolated peripheral blood samples after red blood cell lysis. Cell debris were excluded via appropriate forward (FSC) and sideward scatter (SSC) gating and cell doublets were eliminated using area (FSC-A) versus width (FSC-W) signal intensity. Among CD45^{+} cells, monocytes were gated out of other immune cells (CD3e/CD19/NK1.1/Ly6G – FITC negative) by CD115 (PE-Cy7 positive) and CD11b (APC positive) staining. Monocytes were characterized based on Ly6C (PerCp-Cy5.5) and CD43 (PE) staining

Murine spleen cells The whole spleens were kept in PBS + 10 % FCS directly after isolation and were carefully dissociate with the syringe plunger of a 1 ml syringe by carefully ‘pressing/mashing’ the spleen through a 70 µm cell strainer mesh. The suspension was centrifuged 5 min at 500xg at 4°C and RBC lysis was performed as described above. After RBC lysis the cells were centrifuged 5 min at 500xg at 4°C and resuspended in 1 ml PBS + 10%FCS and filtered again through a 50 µm mesh. Blocking and staining for flow cytometry was performed as described above for murine monocytes. Spleen cells were stained with the anti-CD45 as well as anti CD43 antibody for the positive selection of leukocytes in the cell suspension. LRRK2 staining was performed as described above for human monocytes. LRRK2 expression was analyzed in CD45 and CD43 positive cells via flow cytometry (LSRII, BD).

All FACS data were analyzed with FACS Diva software v6.1.3 (BD) as well as with FlowJo V10 (TreeStar).

Isolation of murine peripheral blood mononuclear cells (PBMCs)

Blood was taken via terminal cardiac puncture. Samples from three mice where pooled and diluted once in PBS and overlaid onto Histopaque-1083 (Sigma). Samples were spun with 1600 rpm for 25 minutes at room temperature without brake. PBMCs were collected at the plasma/Histopaque interface and washed twice with PBS.

Reverse transcription polymerase chain reaction (RT-PCR)

cDNA from murine PBMCs were transcribed from isolated RNA as described above. LRRK2 expression in PBMCs was determined according to the standard PCR protocol version 4.0 provided by The Jackson Laboratories with minor modification. Suggested primers were ordered at Thermo Scientific. Dream Tag PCR Master Mix (2x; Thermo Scientific) was used for the amplification by PCR. Products were visualized on a 2% agarose-gel by staining with ethidium bromide.

Statistical analysis

Statistical analyses were carried out using GraphPad Prism version 6.05 for Windows, GraphPad Software. The values in all figures are presented as mean ± SEM unless stated otherwise. Unpaired t-test was used for unpaired data and paired t-test for paired data from Gaussian distribution. Mann-Whitney test (unpaired data) or Wilcoxon matched-pairs signed rank test (paired data) was used for data from non-Gaussian distribution and all tests for significance were two-tailed. 2-way ANOVA was used as indicated.
