# Supplementary material for: LRRK2 contributes to monocyte dysregulation in Parkinson’s disease
Source: Acta Neuropathol Commun. 2016 Nov 24;4:123. doi: 10.1186/s40478-016-0396-2 (PMC5123218; doi:10.1186/s40478-016-0396-2)
Supplement: Supplementary file 2 — LRRK2 protein expression is significantly upregulated in monocytes from PD patients. (PDF 303 kb) [file 40478_2016_396_MOESM2_ESM.pdf]

LRRK2 contributes to monocyte dysregulation in Parkinson's disease

Corinna Bliederhaeuser[1], Lisa Zondler[1], Veselin Grozdanov[1], Wolfgang P. Ruf[1], David Brenner[1], Heather L. Melrose[2], Peter Bauer[2], Albert C. Ludolph[1], Frank Gillardon[3], Jan Kassubek[1], Jochen H. Weishaupt[1], Karin M. Danzer[1,§]

[1] Department of Neurology, Ulm University, Ulm, Germany

[2] Department of Neuroscience, Mayo Clinic Jacksonville, Jacksonville, FL, USA

[3] Boehringer Ingelheim Pharma GmbH & Co KG, CNS Diseases Research, Biberach/Riss, Germany

[§]Corresponding author: Prof. Karin M. Danzer
Department of Neurology
Ulm University
Albert Einstein Allee 11
89081 Ulm, Germany
karin.danzer@uni-ulm.de
phone: +49 731-500-63049 (office)
fax: +49 731-500-63050

Additional file 2

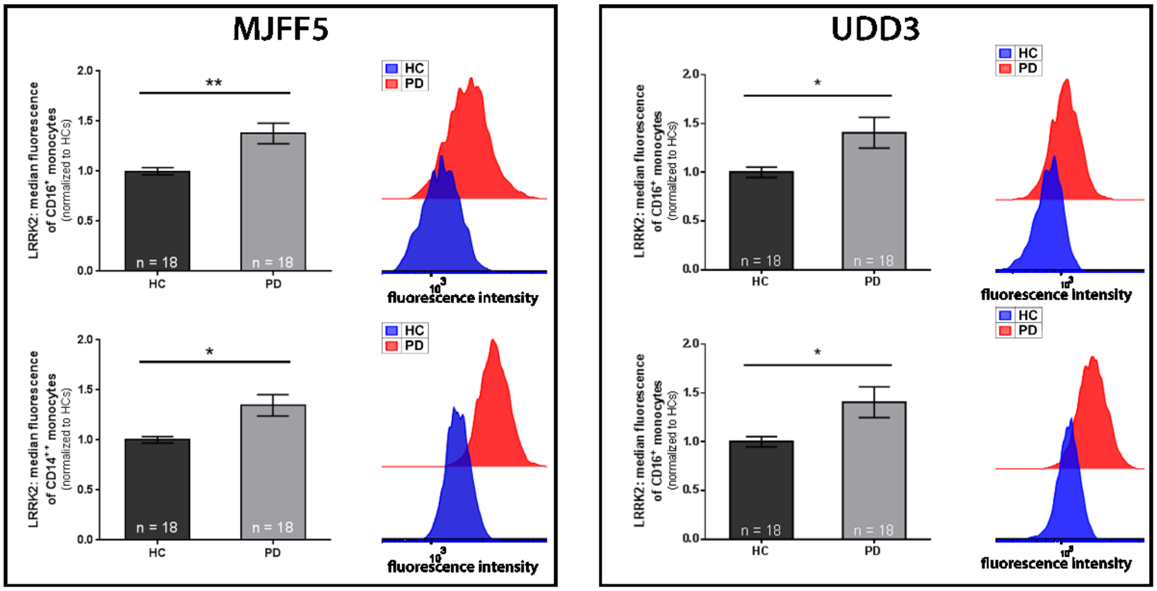

Additional file 2: LRRK2 protein expression is significantly upregulated in monocytes from PD patients Leukocytes from whole blood samples of healthy controls (HC; n=18) and PD patients (PD; n=18) were analyzed by flow cytometry with two additional anti-LRRK2 antibodies to detect LRRK2 protein in the different monocyte subsets. After the cell surface staining as described for human monocytes in Additional file 1, cells were stained intracellularly with monoclonal rabbit-anti-LRRK2 antibodies from abcam, MJFF5(68-7) (left panel) and UDD3 30(12) (right panel), followed by donkey anti-rabbit IgG (clone Poly4064) Alexa Fluor®647 (as further described in Additional file 1). Both monoclonal antibodies reveal similar results of elevated LRRK2 levels in monocytes from PD patients compared to healthy controls as already found by using the anti-LRRK2 antibody from Novus (Fig. 1C). The histograms are representative for the analyzed individuals and display the fluorescence intensity of the anti-LRRK2 antibodies. The higher LRRK2 expression in monocytes of the PD patient compared to healthy control are demonstratively shown in these graphs. Error bars represent mean ± SEM, *p<0.05; **p<0.01; statistical significance was tested with non-parametric testing
